# Enhanced immune response induced by P5 HER2/neu‐derived peptide‐pulsed dendritic cells as a preventive cancer vaccine

**DOI:** 10.1111/jcmm.13343

**Published:** 2017-09-25

**Authors:** Zahra Gholizadeh, Jalil Tavakkol‐Afshari, Amin Reza Nikpoor, Seyed Amir Jalali, Mahmoud Reza Jaafari

**Affiliations:** ^1^ Immunogenetic and Cell Culture Department Immunology Research Center School of Medicine Mashhad University of Medical Sciences Mashhad Iran; ^2^ Department of Immunology Medical School Shahid Beheshti University of Medical Sciences Tehran Iran; ^3^ Biotechnology Research Center Nanotechnology Research Center Mashhad University of Medical Sciences Mashhad Iran; ^4^ School of Pharmacy Mashhad University of Medical Sciences Mashhad Iran

**Keywords:** dendritic Cells, peptide vaccine, pan HLA‐DR epitope peptide, cancer

## Abstract

Dendritic cells are special and powerful antigen‐presenting cells that can induce primary immune responses against tumour‐associated antigens. They can present antigens *via* both MHC‐I and MHC‐II, so they have the ability to stimulate both cytotoxic T lymphocytes and T helper cells. Furthermore, CD8^+^ cytotoxic T lymphocytes require activation by CD4^+^ T cells. This requires a CD4^+^T cell activator molecule, of which PADRE is one of the best. We chose an approach to use both of these important arms of the immune system. We prepared dendritic cells from mouse bone marrow, loaded them with our target peptides (P5 peptide alone or P5 + PADRE), and then injected these pulsed dendritic cells alone or in combination with CpG‐ODN (as adjuvant) into BALB/C mice. After the last boosting dose, mice were inoculated with TUBO cells, which overexpress HER2/neu. Two weeks after the tumour cell injection, immunological tests were performed on splenocyte suspensions, and the remaining mice were evaluated for tumour growth and survival. Our data indicate the formulation that contains PADRE plus P5 loaded onto DC in combination with CpG‐ODN was the most effective formulation at inducing immune responses. Interferon production in CD4^+^ and CD8^+^ gated cells, cytotoxicity rates of target cells and mice survival were all significantly greater in this group than in controls, and all the mice in this group were tumour‐free throughout the experiment. Based on our results and the role of HER2/neu as a candidate in human immunotherapy, this approach may be an effective cancer treatment.

## Introduction

Dendritic cells play critical roles in initiating and modulating adaptive T cell responses 1, 2, 3. They induce T cells *via* their ability to take up, process and present antigens and produce cytokines and chemokines 4, 5. Dendritic cells are the only antigen‐presenting cells (APCs) that are able to prime naïve T cells. Cross‐presentation of antigens *via* DCs is most likely the primary mechanism of CD8^+^ response induction 6, 7. DCs appear in two forms in their life‐times; immature DCs can take antigens, process them and present the antigen‐derived peptides on their major histocompatibility complex (MHC) molecules. Subsequently, DCs change to a phenotypically mature form, which can be distinguished by the increased expression of certain cell surface markers, including CD40, CD80 and CD86 8, 9.

One unique property of DCs is their ability to migrate from environmental sites of pathogen entrance to T cell sites in lymph nodes 10, where they prepare naïve T cells *via* antigen‐specific and costimulator signals. As a result, the immune system can identify invading agent molecules and their pathogenic strengths 11. *In vitro* DCs are derived from CD34^+^ precursor cells or CD14^+^ monocytes 12 using granulocyte–macrophage colony‐stimulating factor (GM‐CSF) 13 and other cytokines, such as IL‐4. These cytokines inhibit macrophage differentiation and induce monocyte‐derived DC production 14.

In addition to using DCs to induce specific immune responses, one well‐known strategy to increase peptide vaccine strength is to induce CD4^+^ T cells that have important roles in CD8^+^ and memory T cell responses. CD8^+^ T cells are especially important for responses to weakly immunogenic antigens such as tumour‐associated antigens (TAAs) 15, 16. One of the most effective molecules used to induce CD4^+^ responses is the pan HLA‐DR epitope peptide (PADRE) 17. PADRE is a universal, non‐specific MHC class II‐restricted epitope able to attach to more than 16 types of common HLA‐DR, I‐A ^b/d^ and I‐E ^b/d^ mouse haplotypes with high affinity. This allows it to overcome the problem of HLA polymorphism 18, 19, 20. It also has shown in clinical trials minimum toxicity 18, 21. Another group of molecules that can improve vaccine immune responses are unmethylated CpG motifs that are used as vaccine adjuvants. CpG motifs are recognized by Toll‐like receptor 9 (TLR9) and increase innate immune responses such as pro‐inflammatory cytokine release and Th1 production. Because of their stability, low cost and ease of production, CpGs are attractive to use in immune system studies 22. CpGs also increase professional APC function and generate both humoural and cellular specific immune responses 23, 24.

Based on our previous study results, the P5 peptide can induce cytotoxic T lymphocyte (CTL) responses in mice bearing HER2‐positive tumours 25. P5 peptide is derived from rat HER2/neu protein (also known as p185 or c‐erb‐B2) with 21 amino acid length (aa 5–25). The murine c‐erbB‐2 shows 93.4% homology at the nucleotide level and 94.8% homology at the amino acid level with rat c‐erbB‐2. Rat HER2/neu is 96% homologous to mouse HER2/neu and 88% homologous to human HER2/neu in overall(1).

ELAAWCRWGFLLALLPPGIAG. Amino acids in boldface type are those in rat HER2/neu, which are different from those in HER2/neu murine sequence 25. The goal of the current study was to overcome the peptide vaccine limitations, such as its weak immunogenicity 26, binding to non‐professional APCs, and rapid degradation by tissue and serum peptidases 27. Here, DCs were prepared *in vitro* from mouse bone marrow stem cells. Target peptides were loaded onto the prepared DCs, and pulsed DCs with or without CpG‐ODN were injected into mice. Immunological *in vitro* tests were performed on mice splenocyte suspensions to assess the immune responses. Mice inoculated with TUBO overexpressing HER2/neu cells were analysed for tumour growth and survival.

## Materials and methods

### Mice

BALB/C, 4‐ to 6‐week‐old female mice were bought from the Pasteur Institute (Tehran, Iran). The mice were kept in the animal house of the Pharmaceutical Research Center (Mashhad, Iran) under controlled conditions of 23°C room temperature, relative humidity of 65% in 12/12‐hr light/dark cycles with free access to water and animal food. All animal experiments were carried out under the approval of the Institutional Ethical Committee and Research Advisory Committee of Mashhad University of Medical Sciences.

### Cell lines

TUBO cells, a cloned cell line that overexpresses HER2/neu, kindly provided by Dr. Pier‐Luigi Lollini (Department of Clinical and Biological Sciences, University of Turin, Orbassano, Italy), were cultured in Dulbecco's modified Eagle's medium (DMEM) and supplemented with 20% foetal bovine serum (FBS). A murine colon carcinoma cell line, CT26, was purchased from the Pasteur Institute and cultured in RPMI‐1640 medium supplemented with 10% FBS.

### Peptides, protein and oligonucleotide

The P5 (ELAAWCRWGFLLALLPPGIAG) and PADRE (AKFVAAWTLKAAA) peptides were synthesized by Peptron Inc. (Daejeon, South Korea). OVA‐FITC protein was purchased from Invitrogen (Carlsbad, CA, USA). CpG‐ODN1826 (5‐TCCATGA**CG**TTCCTGA**CG**TT‐3) was purchased from Mycrosynth (Balgach, Switzerland) 28.

### Generation of DCs

Dendritic cells were generated *via* a 7‐day protocol. Briefly, on day 0 the mouse bone marrow cells were collected and cultured in Iscove's modified Dulbecco's media (IMDM) (Invitrogen) supplemented with 10% FCS (Gibco, Gaithersburg, MD, USA) containing 25 ng/ml of GM‐CSF and 5 ng/ml of IL‐4. On days 3 and 5, cells were seeded in new plates. On day 7, 1 μg/ml of lipopolysaccharide (LPS) was added to the cells and the cultures were incubated at 37°C for 6 hrs. After 6 hrs, the DCs were mature.

### Peptide pulsing of DCs

2.5 μg/ml of each peptide was added to the mature DCs and incubated for 1 hr at 37°C.

### Immunization

After the incubations with peptides, the DCs were washed and counted. 5 × 10^5^ of these DCs containing 0.5 μg of peptide were injected into each mouse. Vaccinations with peptide alone were performed with 100 μg/mouse.

BALB/C mice (Eight per group) were subcutaneously injected three times at 2‐week intervals with the following formulas: buffer (as a control group), CpG, P5, P5 plus CpG, PADRE, PADRE plus CpG, DCs pulsed with P5, DCs pulsed with P5 plus CpG, DCs pulsed with P5 plus PADRE or DCs pulsed with P5 plus PADRE plus CpG.

### ELISpot

ELISpot assays were performed to assess IFN‐ production from splenocytes in response to the different formulas as previously described with mouse ELISpot kits from U‐CyTech (Utrecht, the Netherlands) 29. Briefly, ELISpot plates were coated with anti‐IFN‐ antibody and incubated overnight at 4°C. The following day, splenocyte suspensions of 3 × 10^5^, 2 × 10^5^ and 1 x 10^5^ cells were added to the wells and incubated at 37°C for 24 hrs. Spots were counted with the Kodak 1D software package (version 3.5, Eastman Kodak, Rochester, New York) in triplicate wells and the mean ± S.E.M. was expressed as spot‐forming units (SFUs)/10^6^ splenocytes.

### *In vitro* CTL activity assay

Using *ex vivo*‐expanded splenocytes, *in vitro* CTL assays were performed as described previously 25.

Briefly, TUBO or CT26 (as negative control) cells were resuspended in DMEM with 20% FBS. Calcein AM (Invitrogen) at a final concentration of 12.5 μM was added to the cells and the cells were incubated for 1 hr in the dark at 37°C. Target cells loaded with Calcein AM (1.2 × 10^5^ cells/well) were cocultured with the three different concentrations of splenocytes for 4 hrs at 37°C. The fluorescence intensity was measured at 485 nm of excitation and 538 nm of emission *via* a fluorescent plate reader (FLX 800, Bio‐Tek Instruments Inc. Beverly, MA, USA). The mean percentage of the triplicate wells was calculated by the following formula: [(release by CTL − release by targets alone)/(release by 2% Triton X‐100 − release by targets alone)] ×100.

### Intracellular cytokine assay *via* flow cytometry

Splenocytes (10^6^ cells/ml) in a medium containing 1 μl/ml Golgi Plug™ with 2 μg/ml PMA/ionomycin cocktail were stimulated at 37°C for 4 hrs. Cell surface markers were stained with anti‐phytoerythrin (PE)‐cy5 CD4 and anti‐PE‐cy5 CD8 antibodies and incubated. The cells were then stained with anti‐PE‐IL4 and FITC anti‐IFN‐ antibodies (Cytofix/Cytoperm™; BD Biosciences, San Jose, CA, USA) and analysed by FACSCalibur (BD Biosciences). All antibodies were from BD Biosciences.

### Uptake assay

To assess the protein uptake by prepared DCs, mature DCs were incubated with 2.5 μg/ml of OVA‐FITC at 37 and 4°C (as a control) for 1 hr. Antigen uptake by DCs was analysed by FACS and the mean fluorescence intensity (MFI) measured.

### *In vivo* challenge of immunized mice with TUBO cells

To study tumour growth, 2 weeks after the last boost, vaccinated mice were inoculated subcutaneously with 5 × 10^5^ TUBO cells in their right flanks. The tumours were measured with a calliper and volumes were calculated using the formula (a × b × c) × 0.5 mm^3^ 25.

Mice were monitored for 100 days after injection.

### Statistical analysis

The results were analysed using one‐way anova with GraphPad Prism version 6 (GraphPad software, San Diego, CA, USA). In the case of a significant *P* value, the multiple comparison Tukey test was used to compare the means of different formulas.

The log‐rank test (GraphPad Prism, version 6) was used to analyse mice survival. When *P* < 0.05, results were considered significant.

## Results

### Phenotype of mature and immature DCs

**Figure 1:**
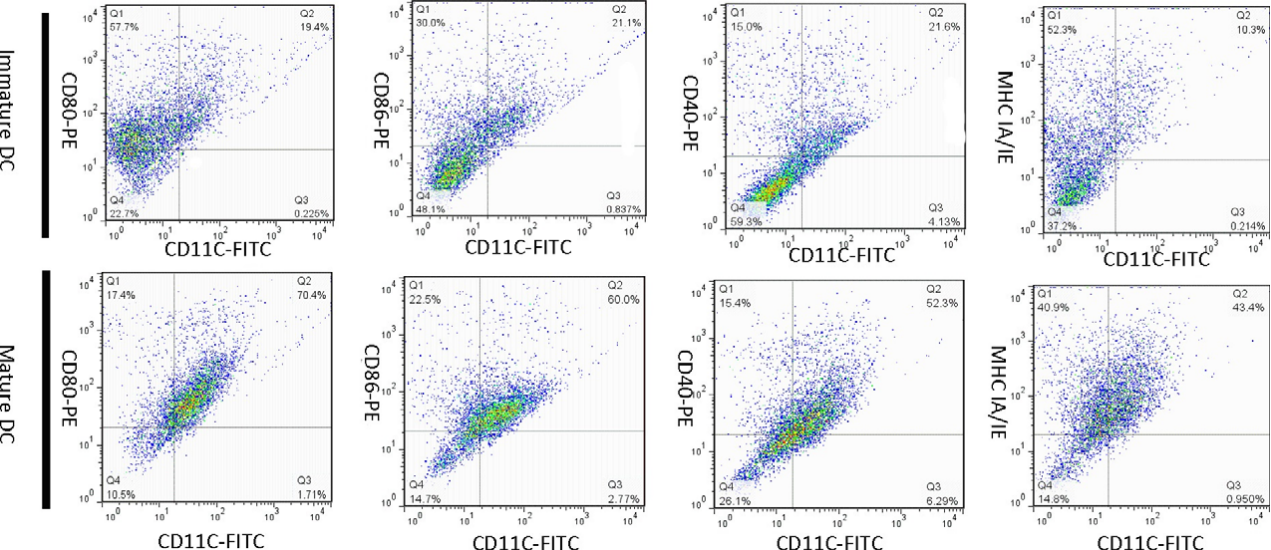
CD11c, CD40, CD80 (B7‐1), CD86 (B7‐2) and MHC IA/IE expression on immature and mature DCs. Expression of these markers is greater in mature than in immature DCs. Dendritic cells were generated from mouse bone marrow and matured by the addition of LPS. The dot plot represents cell percentages in the mature and immature states.

On day 5 and 7, DCs were harvested and characterized using DC surface marker antibodies (anti‐CD11c, anti‐CD40, anti‐CD80, anti‐CD86 and anti‐IA/IE). As shown in Figure 1, MHC and costimulatory molecule expression are greater in mature than in immature DCs and most of the DCs were mature after LPS stimulation.

### Uptake assay

**Figure 2:**
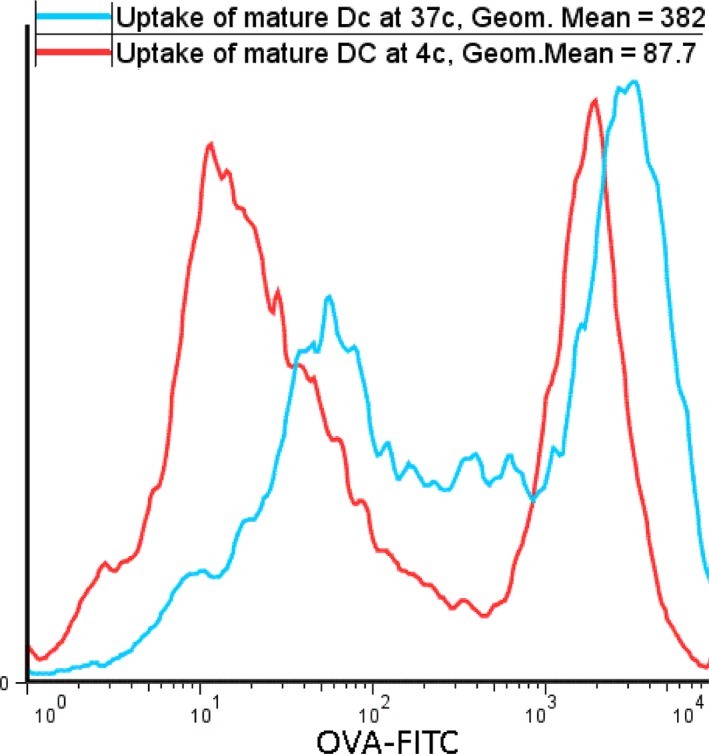
Uptake of OVA‐FITC by DCs. The uptake assay was performed to analyse the endocytic ability of prepared DCs. Dendritic cells took up more OVA‐FITC 37°C than at 4°C. The fluorescent intensity corresponds to the uptake.

The endocytic capability of prepared DCs at 37°C was greater than at 4°C and the geometric means at 37 and 4°C were 382 and 87.7, respectively (Fig. 2). Overall, the generated DCs took up peptides with high efficacy.

### Intracellular cytokine assay *via* flow cytometric analysis

**Figure 3:**
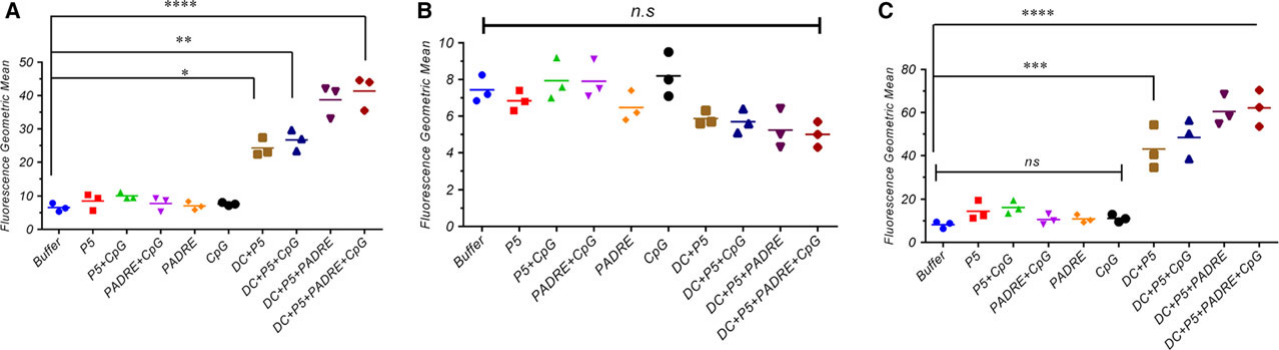
Geometric mean fluorescence intensity (MFI) of IFN‐γ in gated CD4^+^ cells (**A**), IL‐4 in gated CD4^+^ cells (**B**) and IFN‐γ in gated CD8^+^ cells (**C**). Cytokine profile frequency was analysed by FACS analysis to determine the immune response rate. Splenocytes were stained by anti‐CD4 and anti‐CD8 antibodies. After stimulation with PMA and ionomycin, cells were stained with anti‐IFN‐γ and anti‐IL‐4 antibodies. Data are expressed as the mean ± S.E.M. (*n* = 3). Results were analysed by one‐way anova, and statistically significant differences are designated as follows: ns: *P* > 0.05, *P < 0.05, **P < 0.01, ***P < 0.001, ****P < 0.0001.

To analyse the effect of the different formulas on induction of CD4^+^ and CD8^+^ T cells, their cytokine profiles were assessed. Induction of IFN‐γ by CD8^+^ cells by P5 and PADRE peptides alone or with CpG was not significantly different from control. However, IFN‐γ release was significantly increased when the peptides were loaded onto DCs. Immunization with P5 plus PADRE‐loaded DCs along with CpG stimulated IFN‐γ secretion by CTLs, and significantly more IFN‐γ was detected in gated CD8^+^ cells than in the control or other treatment groups (*P* < 0.001) (Fig. 3C). A similar significant effect was observed in the IFN‐γ‐producing CD4^+^ cells (Fig. 3A) (*P* = 0.0005). In contrast, IL‐4 production was not affected in CD4^+^ cells, indicating that humoural immunity was not significantly induced in the vaccinated groups (Fig. 3B).

### *In vitro* IFN‐γ assay by ELISpot

**Figure 4:**
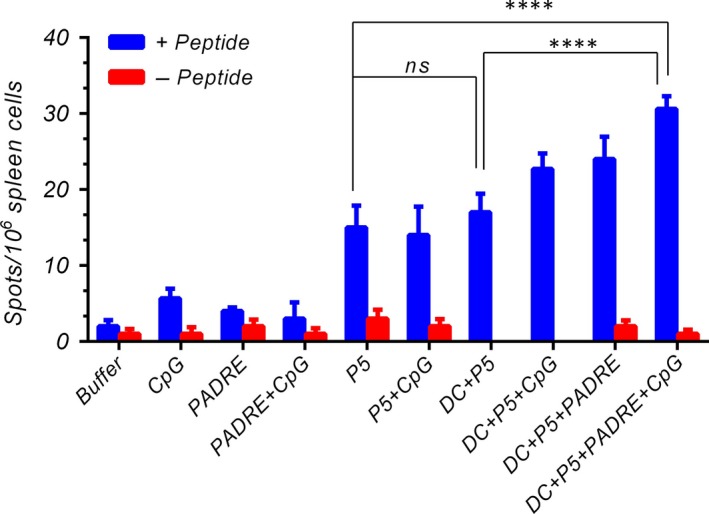
In vitro IFN‐γ production was analysed in vaccinated mice 2 weeks after the last booster by ELISpot assay. Splenocytes were harvested and treated with (+peptide) or without peptides (‐peptide) and the IFN‐γ secretion was determined. The data indicate the mean ± S.E.M. (*n* = 3). Statistical significances were designated as follows: ns: P > 0.05, *P < 0.05, **P < 0.01, ***P < 0.001, ****P < 0.0001

P5 alone on DCs caused no statistically significant increase in IFN‐γ; however, DCs loaded with both P5 and PADRE peptides and co‐administered with CpG resulted in significantly greater IFN‐γ production from T cells than controls, which supports the flow cytometry result (*P* < 0.001) (Fig. 4). However, the level of IFN‐γ in pulsed DCs with peptides without CpG was slightly less than with this formulation with CpG. Our data indicate that the immune responses in mice vaccinated with DCs loaded with both of two targeted peptides are significantly greater than peptides alone or DCs loaded with P5 alone.

### Cytotoxicity assay

**Figure 5:**
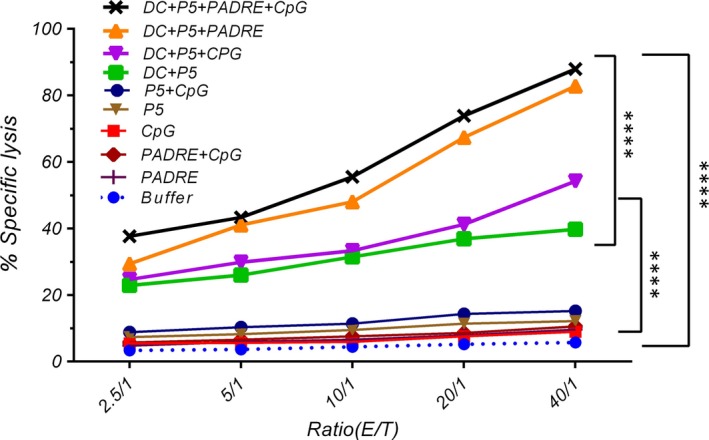
Induction of specific CTL potency for tumour cell killing by the different formulations was assessed by *in vitro* CTL activity assay. Five different effector to target cell ratios were tested. HER2/neu‐expressing TUBO cells and CT26 cells (as a negative control) were labelled by Calcein AM and co‐incubated with different ratios of splenocytes. The data are expressed as means ± S.E.M.s (*n* = 3). E: effector cells and T: target cells. One‐way anova was employed and statistically significant differences are shown as follows: ns: *P* > 0.05, *P < 0.05, **P < 0.01, ***P < 0.001, ****P < 0.0001.

The specific responses of CTL cells against cancer cells in groups immunized with pP5 or P5 + CpG or PADRE and PADRE + CpG were no greater than those of the control group (Fig. 5). As expected, vaccination with DCs + P5 + PADRE + CpG was best able to induce CTLs to recognize and kill the TUBO cells (*P* < 0.001). At all E/T ratios, the per cent of TUBO cell killing by CD8^+^ cells was greatest with this formula. CTLs did not kill HER2/neu‐negative CT26 cells, indicating that the cytotoxic response was antigen‐specific.

### Tumour size and survival

**Figure 6:**
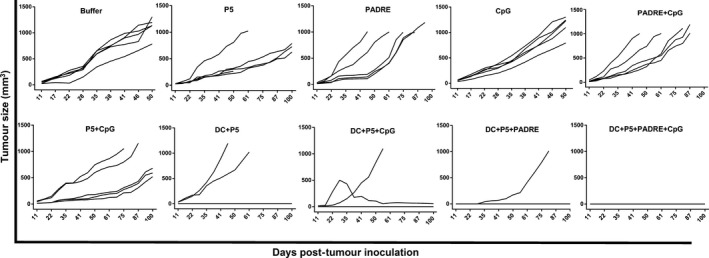
Results of the formulations on TUBO tumour growth in mice. Two weeks after the final boosting dose, TUBO cells were injected into mice (*n* = 5). Tumours were measured and volumes recorded weekly. The values are means. Mice were monitored for 100 days. Data were analysed by the two‐way anova test and statistical significances were designated as follows: Ns: *P* > 0.05, *P < 0.05, **P < 0.01, ***P < 0.001, ****P < 0.0001.

**Figure 7:**
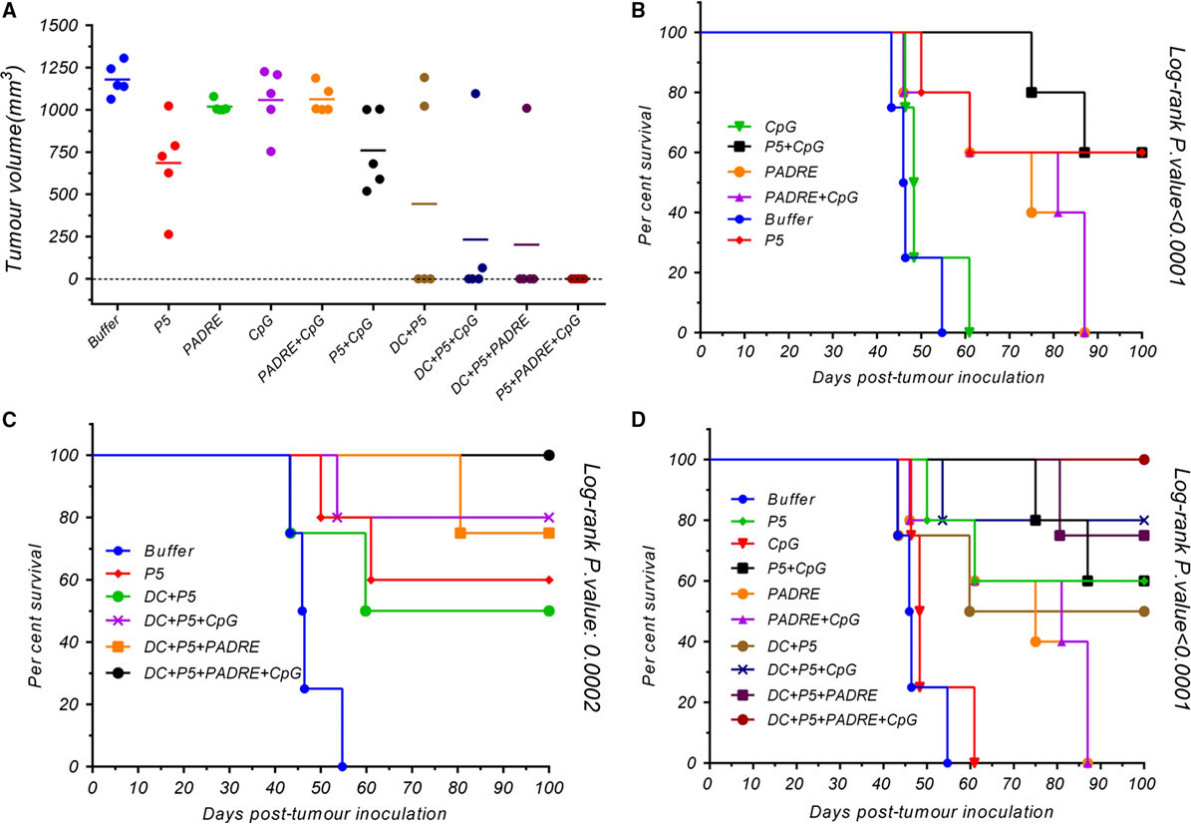
The tumour mass scale based on tumour size in inoculated mice on their last survival days (**A**), and their survival (**B**,** C** and **D**) were monitored by the multiple comparison log‐rank (Mantel–Cox) test. Statistical significances were designated as follows: ns: *P* > 0.05, *P < 0.05, **P < 0.01, ***P < 0.001, ****P < 0.0001.

Pulsed DCs showed significantly greater antitumour activity than the control group (*P* < 0.001). P5 or PADRE alone as a peptide vaccine with or without CpG inhibited tumour growth significantly less than the same peptide loaded on DCs (Figs 6 and 7A).

The DC + P5 + PADRE + CpG formulation completely inhibited tumour growth. All the mice vaccinated with this formulation were tumour‐free throughout the study, while no mice immunized with peptides alone that survived until day 100 were tumour‐free.

Table 1 displays the different formulations and their corresponding times to reach end‐points (TTE), percentages of tumour growth delay (%TGD) and median survival times (MST). Median survival times were undefined in groups in which most or all the mice survived the duration of the experiment (Table 1).

**Table 1:** Beneficial effect of each formulation in immunized mice

Groups	TTE (days ± S.D.)^a^	%TGD^b^	MST(day)^c^
Buffer	47.59 ± 4.97	–	46.18
P5	77.11 ± 26.65	29.01	Undefined
PADRE	92.36 ± 11.33	20.88	75
CpG	50.98 ± 6.69	6.66	48.3214
PADRE + CpG	69.96 ± 17.73	21.76	81
P5 + CpG	92.36 ± 11.33	40.74	Undefined
DC + P5	75.80 ± 12.96	37.22	64.39
DC + P5 + CpG	90.72 ± 6.89	47.55	Undefined
DC + P5 + PADRE	95.16 ± 0.00	49.99	Undefined
DC + P5 + PADRE + CpG	100.00 ± 0.00	52.41	Undefined

a Time to reach end‐point.

b Tumour growth delay (in comparison with buffer group).

c Median survival time.

Dendritic cells loaded with P5 + PADRE + CpG inhibited tumour growth completely (TGE = 52%) and increased mice survival times (TTE = 100) more effectively than controls. Mice vaccinated with DCs pulsed with P5 + PADRE with or without CpG had longer life spans than control and peptides alone or with CpG (Fig. 7B–D). The *in vivo* study demonstrates that DC + P5 +  PADRE + CpG had a powerful prophylactic effect on tumour growth, which correlated with mice survival (Fig. 6).

## Discussion

In this study, we used a long multi‐epitope peptide, a CD4^+^ epitope peptide, an adjuvant and DCs as an appropriate delivery system to induce immune responses. As we expected, when the P5 HER‐2/neu‐derived peptide was loaded onto DCs, tumoural immune responses were greater, both *in vitro* and *in vivo*, than when free peptide formulations were used. Moreover, PADRE peptide, as a CD4^+^ T cell activator, remarkably improved the responses generated by P5 peptide loaded DCs, and CpG increased the immune responses.

Peptide vaccines in cancer immunotherapy have been shown to have some advantages 25; however, they also have limitations 17. One limitation is the specificity of peptide epitopes for binding to MHC molecules. To overcome to this problem, we used the P5 peptide, which are 21 amino acids long. Because of its size, P5 contains several epitopes that can activate more T cell colonies and enhance immune responses 30. Our finding is consistent with an earlier study that showed that the HPV16 synthetic long peptide (HPV16‐SLP) as a vaccine in therapy of patients with advanced or recurrent HPV16‐induced gynaecological carcinoma induced CD4^+^ and CD8^+^ T cell responses against HPV16 31.

The second limitation is in finding the optimum delivery system for antigens. Dendritic cells are among the best for this purpose and mature DCs have an important role in the induction of strong immune responses.

Several studies have shown that DC maturation is one of the most important steps in vaccination protocols. Mature DCs are more potent than immature ones in eliciting antitumour responses, likely due to their ability to present TAAs on MHC molecules and up‐regulate co‐stimulatory molecules 32. The possible reason why pulsed DCs worked well *in vivo* is because of their immigration to lymph nodes. It has been shown that migration of antigen‐loaded DCs to lymph nodes is related to their maturation 33. In our study, cultured, peptide‐pulsed DCs grown in the presence of GM‐CSF and IL‐4 induced greater protection against TUBO tumours than a free peptide vaccine. In support of our results, previous studies demonstrated that GM‐CSF is necessary for DC differentiation and function 34, 35, increases the immune response *via* DC activation and increases their migration to lymph nodes 36. Provenge is also a DC vaccine that contains autologous DCs pulsed with a fusion protein consisting of prostatic acid phosphatase (PAP) and GM‐CSF. PAP is a peptide expressed on 95% of human prostate, brain, testicular, spleen and heart cells 37. In our study, mature DCs induced immune responses in all *in vivo* and *in vitro* tests. Previous studies reported that mature or differentiated DCs are the only APCs capable of activating naïve CD8^+^T lymphocytes *in vivo* 38, 39 due to their ability to present exogenous antigens, such as TAA, on MHC‐I 7, 40, 41, 42. In a clinical study de Vries and colleagues compared the capacity of mature and immature DCs to induce immune responses in stage IV melanoma patients. They observed tumour regression in patients receiving mature DCs due to expression of stimulatory molecules, particularly when TAAs with low immunogenicity were presented 43. In another study, autologous DCs, when fused with the allogeneic colorectal carcinoma cell line COLM‐6, induced both CD4^+^ and CD8^+^ T cells and CTL responses that break down autologous colorectal carcinoma cells 2. In another study, monocyte‐derived human DCs pulsed with purified full‐length wt‐P53 stimulated P53‐specific CTLs *in vitro* 4.

Using a delivery system targeting dendritic cells can act as an effective strategy in cancer vaccine.

In a new study, Thi Tran and colleges showed the B subunit of the Shiga toxin (STxB) can be used as a vector that targets dendritic cells. This vector after coupling to different tumour antigens induces specific CD8^+^ T cell responses. This research group finding also mentioned that STxB is effective in human dendritic cell cross‐presentation 44. Another important issue is the role of CD4^+^ T cells in CD8^+^ T cell activation and function. Due to their key role in the development of activated and memory CD8^+^ T cells 15, design of a procedure for CD4^+^ T cell production in immunotherapy could be very useful. Our previous study indicated that PADRE plus long peptides improve immune responses 45; therefore, in the current study, we used PADRE as a CD4^+^ T cell activator plus P5 to enhance specific antitumour responses. Loading PADRE plus P5 onto DCs significantly boosted the immune response. This result is consistent with other work that showed that Th cells play a critical role in CTL induction 46. One explanation for this result is that addition of PADRE causes IL‐2 secretion by CD4^+^ Th‐APCs 47. D'Souza *et al*. showed IL‐2 can enhance expansion efficacy of CTLs 48. Also, transferred CD4^+^ Th cells can promote CD8^+^ CTL expansion and function and this assistance is mediated by IL‐2 49, 50. Another possible reason is due to the costimulatory role of CD80. Umeshappa *et al*. reported that CD80 costimulatory signalling on CD4^+^ Th‐APCs affects CD8^+^ CTL responses 47. In addition, interaction between CD28 and CD80 has been shown to be necessary for T cell proliferation and activation 51.

Although not well characterized, CD4^+^ Th cells have been shown to inhibit tumour growth 52. They also have an indirect effect *via* strengthening CD8^+^ cell responses and attracting and activating macrophages and NK cells 53, 54. Schuurhuis *et al*. showed that CD4^+^‐dependent inhibition of tumour growth in the absence of CD8^+^ cells may also have a tumour‐protective effect 55. Our data demonstrate that PADRE increases the CD4^+^ Th1 subset population, and as a consequence, the immune response against TUBO tumour cells was greater than that of other groups and control.

Wang *et al*. showed that primary CD8^+^ T cell responses to presented antigens *in vivo via* peptide‐pulsed DCs depend on CD4^+^ T cell assistance 56. In our study, loading PADRE onto DCs resulted in a significantly greater immune response than PADRE plus P5 peptide without DCs. In fact, simultaneous use of DCs and PADRE enhanced the effect of each of them. The reason may be due to the CD40‐CD40L interaction, which has an important role in CD4^+^ Th‐mediating function for CD8^+^ T cell responses. DCs present antigens and activate CD4^+^ T cells *via* CD40 costimulatory molecules. When DCs stimulate CD4^+^ cells, these cells also activate DCs *via* CD40L and cause DCs to induce CD8^+^ responses 57, 58, 59. Furthermore, when a potent inflammatory stimulus is not present, DCs must interact with CD4^+^ T cells to induce strong CD8^+^ T cell responses 58.

Another problem with peptide vaccines, which we tried to overcome, is the need for a suitable adjuvant that is able to stimulate immune responses in general. CpG‐ODN has been demonstrated to be an effective adjuvant both *in vitro* and *in vivo* 60, 61, 62. This synthetic, short, single‐stranded oligonucleotide activates Th1 responses *via* interaction with TLR9, and due to inhibition of Th2 responses by Th1 cytokines, including IFN‐γ and IL‐12; this motif is useful in cancer immunotherapy 63. Although in our study the immune responses were not significantly different in the presence or absence of CpG, it could increase the immune response against tumour growth. CpG‐ODN also has a direct stimulation effect on activation and maturation of DCs and macrophages, which result in the production of Th1‐like cytokines 64 seen in our study.

Another critical consideration in immunotherapy is the vaccine dosage. We achieved an immune response with a much lower concentration of peptides on our DC‐treated mice than on peptide‐alone‐treated mice (0.5 μg/mouse *versus* 100 μg/mouse), which could be important in pharmaceutical marketing. One possible reason for the high peptide concentration requirement is that in the latter, the peptides may be removed by other cells, such as macrophages, which are not able to initiate immune responses and activate naïve CD8^+^ T cells. Another possible reason is that removal of peptides by DCs *in vivo* may cause tolerance instead of immune stimulation because the DCs may remain immature and unable to present antigens 65.

In addition to all these, combination therapy against HER2/neu can be applied using monoclonal antibodies together to enhance immune response and survival rate. To reach this goal, Linch SN and colleges demonstrated dual anti‐aOX40 (anti‐CD134)/aCTLA‐4 (anti‐cytotoxic T lymphocyte‐associated protein 4)mAb in combination with anti‐DEC‐205 (dendritic and epithelial cells, 205 kD)–HER2. They analysed that following this immunotherapy, Th2‐cytokine generation by CD4^+^ was reduced and IFN‐γ production by CD4^+^ and CD8^+^ was enhanced. The combination therapy was associated with tumour‐free survival, gathering of effector T cells in tumour site, stimulated as well as reversed T cell anergy resulting in tumour regression 66.

One of the goals of our study was to address some of the limitations of present cancer vaccines. The use and strengthening of the immune system *via* DCs and CD4^+^ T cell activation, which are suppressed by cancer cells in many cases, can be an effective strategy in cancer immunotherapy.

## Conflict of interest

The authors declare that there is no conflict of interest.
